# Discourses on smoke-free policies on Dutch Twitter: A social network analysis

**DOI:** 10.1177/20552076251325583

**Published:** 2025-03-14

**Authors:** Roel O Lutkenhaus, Abel Meijberg, Famke JM Mölenberg, Jasper V Been, Martine PA Bouman

**Affiliations:** 1 640003 New Momentum, Rotterdam, The Netherlands; 2 Center for Digital Inclusion, 159204 Faculty of Behavioral, Management and Social Sciences (BMS), University of Twente, Enschede, The Netherlands; 3 Department of Public Health, 6993 Erasmus MC, University Medical Center Rotterdam, Rotterdam, The Netherlands; 4 Division of Neonatology, Department of Paediatrics, 97759 Erasmus MC Sophia Children's Hospital, University Medical Center Rotterdam, Rotterdam, The Netherlands; 5 Center for Media and Health, Gouda, The Netherlands; 6 Department of Media and Communication, Erasmus School of History, Culture and Communication, 6984 Erasmus University Rotterdam, Rotterdam, The Netherlands

**Keywords:** Social network analysis, tobacco, smoke-free policies, Twitter, discourse

## Abstract

**Objectives:**

In highly mediatized societies, online discourses may contribute to whether novel smoke-free policies become a success. This study analyses Dutch public discourses about smoke-free policies on Twitter, which has been known as X since 2023.

**Methods:**

We analyzed messages about smoke-free policies published by Dutch Twitter users from January 2020 until 31 March 2021. We used search queries designed to find tweets that shed light on health, legislative, and emotional aspects of smoke-free policies and used a mixed-method approach that combined social network analysis, text mining, and qualitative inquiry to identify online communities and understand what they are saying.

**Results:**

We found 45,636 Twitter messages that mostly responded to news about smoke-free policies and tobacco legislation. We identified two larger news communities and five niche communities of which the *Health Care*, *Vaping Lobby,* and *Anti-Establishment* communities were the most substantively engaged. The *Health Care* community focused on spreading health information and exposing the tobacco lobby. The *Vaping Lobby* community pushed vaping as a healthy alternative to smoking. The *Anti-Establishment* community connected smoke-free policies with alleged oppression of civil liberties such as driving cars, eating meat, and COVID-19 measures.

**Conclusions:**

Smoke-free policies are frequently discussed by communities that each approach the topic from their own perspective. Anti-establishment sentiment may pose a threat to implementing smoke-free policies, especially if the *Vaping Lobby* is to align its argumentation with the anti-institutional narratives. The approach presented in this study can strengthen socio-ecological approaches that aim to account for public debate on social media in implementing new policies.

Across the world, countries are now implementing policies that create smoke-free environments in outdoor public spaces (e.g. parks, beaches) and in (semi)private spaces (e.g. cars, balconies), which have been shown to contribute to improved health outcomes.^1–6^ The World Health organization (WHO) has stated that involving civil society is central to achieving effective tobacco control legislation^7^ (2009). After all: when policies are formally in place but not supported followed or enforced properly, the prospective health gains for citizens are unlikely to be realized. Research shows that the interaction between technology and communication practices may influence opinions and attitudes, and—due to the deeply mediatized nature of contemporary societies—may ultimately impact the direction in which societies evolve.^8–10^ To successfully develop and implement smoke-free environments, it may therefore be expedient to understand the contexts that contribute to shaping people's attitudes and opinions around the topic, including the debate on social media platforms.

Media behaviors have diversified over the past two decades: audiences have moved to online environments where they gather around specific niche interests.^9,11,12^ In online communities, like-minded audiences interact with each other on their favorite topics (e.g. music, food, fashion, public issues like smoke-free environments) by consuming media content as well as creating their own.^13–15^ In the Netherlands, more than 85% of the population aged 12 or older uses social media platforms; 50% actively post pictures, music, or text; and 7,7% publicly share their opinion about politics and societal topics.^16^ Sometimes, members of online communities collectively use their voices to relate to a *social domain*, for example by raising awareness for an issue such as the need for smoke-free environments, while online communities can also have conflicting interests and relate to social domains with rival interests.^9,17^ Collective media engagement influences the public news agenda,^18^ affects flows of information,^9,19^ and is associated with renegotiations of social norms, meaning, and culture.^10,20,21^

If we follow a socio-ecological approach,^22^ considering the highly mediatized context in the Netherlands, online discourse can play a role in whether novel smoke-free policies can be implemented successfully. This article focuses on discourses on X, formerly known as Twitter: a micro-blogging platform that—at that time—was mostly used by journalists, politicians, communications professionals, scientists, and avid news followers. At the time when this study was conducted, X was still known as Twitter and we will therefore refer to it as such. In 2020, about 88% of the Dutch population aged 12 years and over used one or more forms of social media.^23^ According to the National Social Media Study, 3.5 of 17.5 million people (20%) in the Netherlands were using Twitter in 2022, and 1.4 million people (8%) in the Netherlands were using it daily.^24^ At that time, Twitter often followed the dynamics of the mainstream news agenda and influenced this agenda itself.^18,25^ But Twitter had also been associated with a growing anti-establishment movement and coordinated misinformation campaigns.^11,26–28^To achieve an understanding of the online discourse on Twitter around smoke-free environments, we aimed to:

1. Identify online communities that regularly engage with the topic of smoke-free environments; and
2. map the alignment of online communities with social domains when talking about smoke-free environments
3. probe the level of support for novel smoke-free policies among various communities of like-minded audiences.

## Method

This study was part of a Dutch research consortium that focused on novel smoke-free policies across the world, their legal latitude and effectiveness, and how they can be implemented in the Netherlands.^2,29–33^ As part of a larger analysis of the public discourse about smoke-free policies on social media (i.e. Twitter, YouTube, and the Open Web) that aimed to contribute to a comprehensive understanding of the societal context, this study specifically focused on the public debate on Twitter between 1 January 2020 and 31 March 2021.

### Search queries

In collaboration with public health and law researchers, we generated a list of seven domains related to smoke-free policies: smoke-free, tobacco, policy, location, stakeholders and target groups, human rights, and support. For each category, we identified associated keywords and their synonyms (see Table 1). We created search queries to find Twitter messages that included any of the keywords from the “smoke-free” category and/or “tobacco” category, in combination with a keyword from at least one of the other five categories.

**Table 1.** Keyword categories.

Category	Keywords
Smoke-free	rookvrij, tabaksvrij^b^
Tobacco	rook, roken, tabak, sigaret, sigaar, sigaren, peuk, saffie, saffen, paffen, paffer, roker, shag, sjekkie^c^
Policy	beleid, regel, reguleren, wetten, wetgeving, verbod, verbied, ‘mag niet’, illegaal, strafbaar, boete, accijns, prijs, prijzen, kost, ontmoedig, beperk, maatregel^d^
Location	zone, gebied, bushalte, school, omgeving, terrein, ziekenhuis, ‘publieke ruimte’, ‘openbare ruimte’, terras, restaurant, auto, huis, woning, appartement, balkon, café, bar, horeca, strand, OR vliegveld, station^e^
Stakeholders and target groups	overheid, kind, jeugd, jongere, peuter, kleuter, baby, minderjarig, ouder, personeel, medewerker^f^
Human rights	welzijn, gezondheid, ontwikkeling, ‘schone lucht'^g^
Support	steun, mening, opinie, hinder, overlast, recht, autonomie, ‘vrij keuze’, betutteling, ‘in strijd met’^h^

### Data retrieval and processing

When the study was conducted, Twitter offered academics the opportunity to request access to their Research API v2 that—in contrast to the public API—allowed us to search the full archive of Twitter messages.^34^

#### Retrieval of messages

We requested and were granted access and used R-scripts to send our search queries to the API to collect Twitter messages published between 1 January 2020 and 31 March 2021.^34^ This timeframe reflected the purpose of the study well, which was to inform stakeholders such as policy-makers about the state of the public debate on Twitter. We also collected chains of retweets, quotes, and replies associated with the messages we initially found, reflecting the wider context in which discussions about smoke-free policies and environments occurred. Therefore, we will refer to the messages we initially found from here on as tweets belonging to the *conversation level*, and to the messages that we found by following chains of retweets, quotes, and replies as the *conversation's context* level. The authors of tweets on both of these levels will be referred to as *active users*.

To retain only the messages that were actually about smoking and/or smoke-free policies, we filtered the tweets for relevance. First, we created a list of the most common words and word combinations across the dataset to get a better sense of the topics reflected most frequently in the data set. We then filtered the tweets to not include messages about themes beyond our interests (see Table 2 for the specific filters), as well as to retain only messages that were written in the Dutch language.

**Table 2.** Keywords used to filter tweets.

Keywords	Translation
hout, kachel, brand, hulpdienst, melding, bbq, biomassa, avondklok, stoken, vuur, vlammen, corona, and covid	wood, stove, fire, emergency service, barbecue, biomass, curfew, to heat, flames, corona, and covid
‘celine’ combined with ‘vermist’, ‘rook’ combined with ‘opgaan’, ‘sigaar’ combined with ‘doos’	‘celine’ (name) combined with ‘missing’ ‘smoke’ combined with ‘to ascend’ (signifying the Dutch colloquialism of something ‘going up in smoke’), cigar comnbined with ‘box’ (signifying the Dutch colloquialism of ‘gifting a cigar from ones’ own box’)

#### Retrieval of followers and followees

For all the active users, we retrieved all *followers* (users subscribed to authors’ timelines) and *followees* (users that authors are subscribed to), reflecting the active users’ *interests and audiences*. These users did not actively tweet about smoke-free policies (from here on referred to as *inactive users*), yet their connectedness does provide relevant information on the wider alignment of the active users. As we aimed to understand the interests and audiences that active users had *in common*, we retained 10% of the inactive users who were most closely connected with the authors.

**Figure 1.**
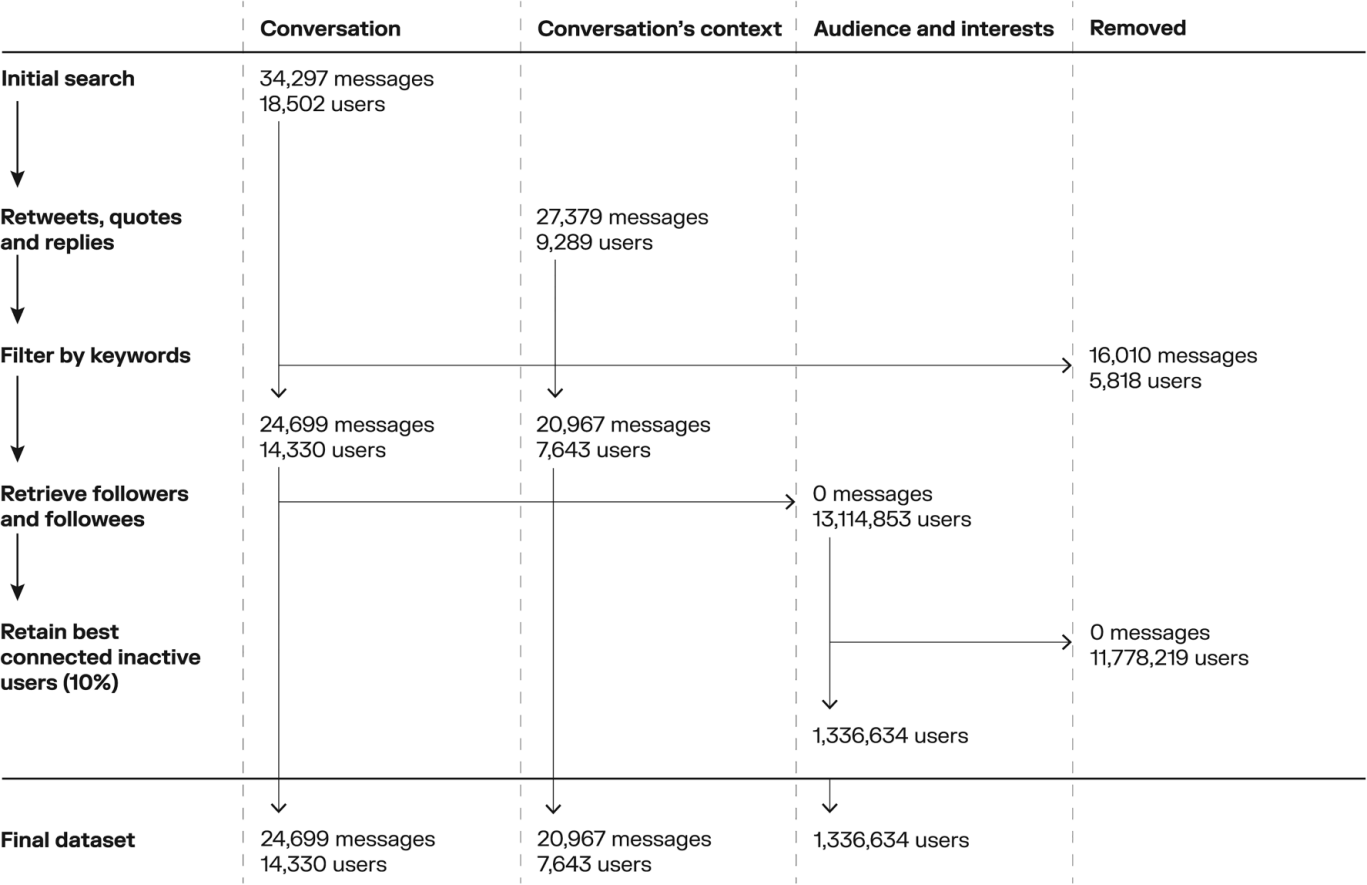
Data retrieval and processing.

We retrieved the profile descriptions (also known as “bio”) for the remaining users. Figure 1 provides an overview of the data retrieval process.

### Data analysis

Once the data were collected and filtered, we pseudonymized all the user handles and persistently stored the data in a Neo4j database, which is a graph database suitable for performing sophisticated network algorithms.^35^ Twitter messages and connections between users that fell beyond the scope of our study were permanently deleted. We analyzed the final data set using a combination of network analysis, text mining techniques, and qualitative inquiry. This was an iterative process comprising three steps aimed at contextualizing quantitative results with qualitative insights.

#### Network analysis to detect communities and identify patterns

First, we detected communities of closely connected Twitter users using the Neo4j Graph Data Science Library's implementation of the Louvain algorithm.^36,37^ This algorithm aims to find communities by iteratively computing a modularity score that measures the relative density of connections inside communities compared to the relative density of connections outside communities. Finding the highest possible value theoretically produces the best possible grouping of the users in the network. Users can only be assigned to one community.

**Figure 2.**
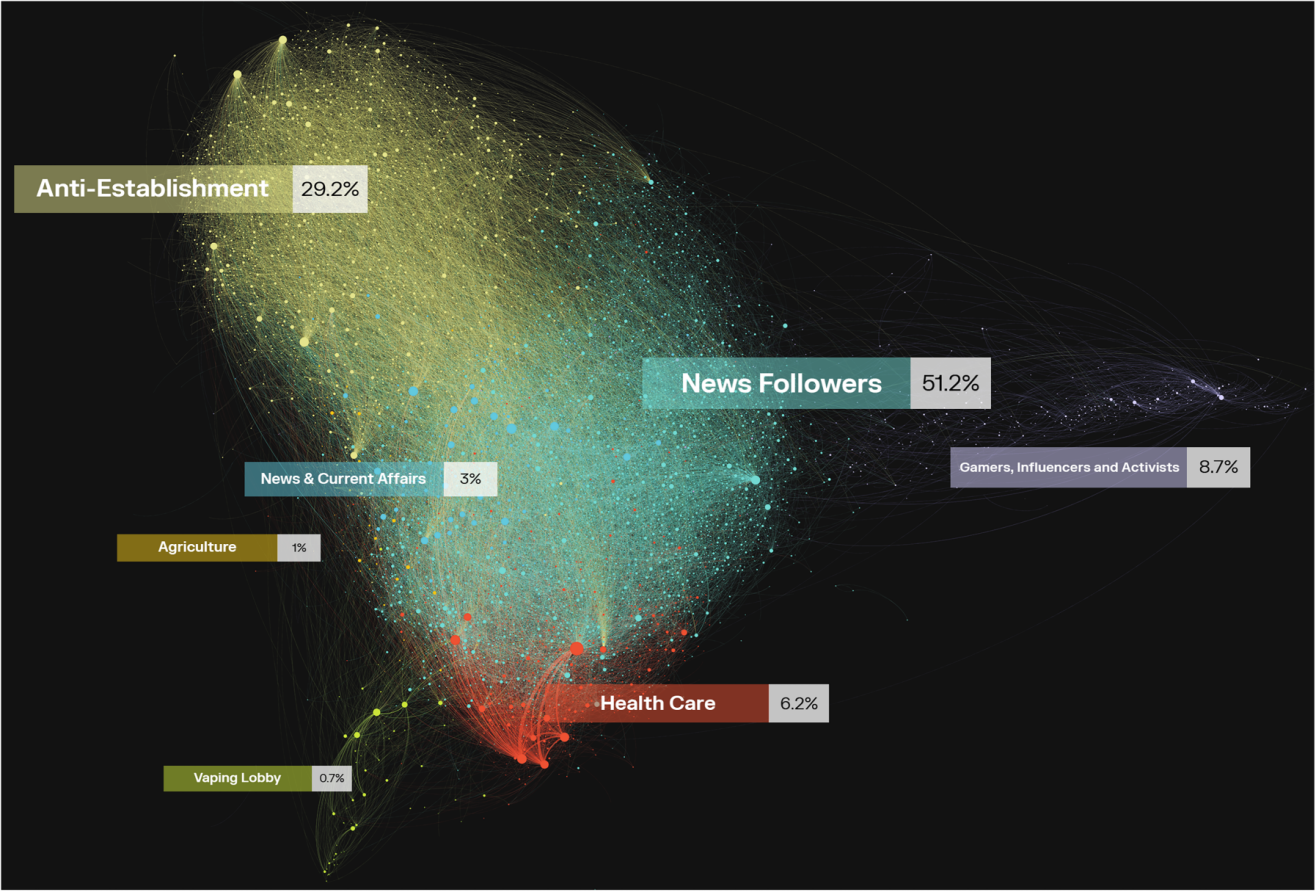
Visualization of the network of active Twitter users.

Note: The dots are Twitter users and the size of the dots express the number of tweets by the users in the data set (meaning that inactive users are invisible). The lines signify mentions (including replies, quotes, and retweets) and the colors show to which community the users belong. This visual only shows active users. The labels include the communities’ names and the percentage of tweets that each community published.

**Figure 3.**
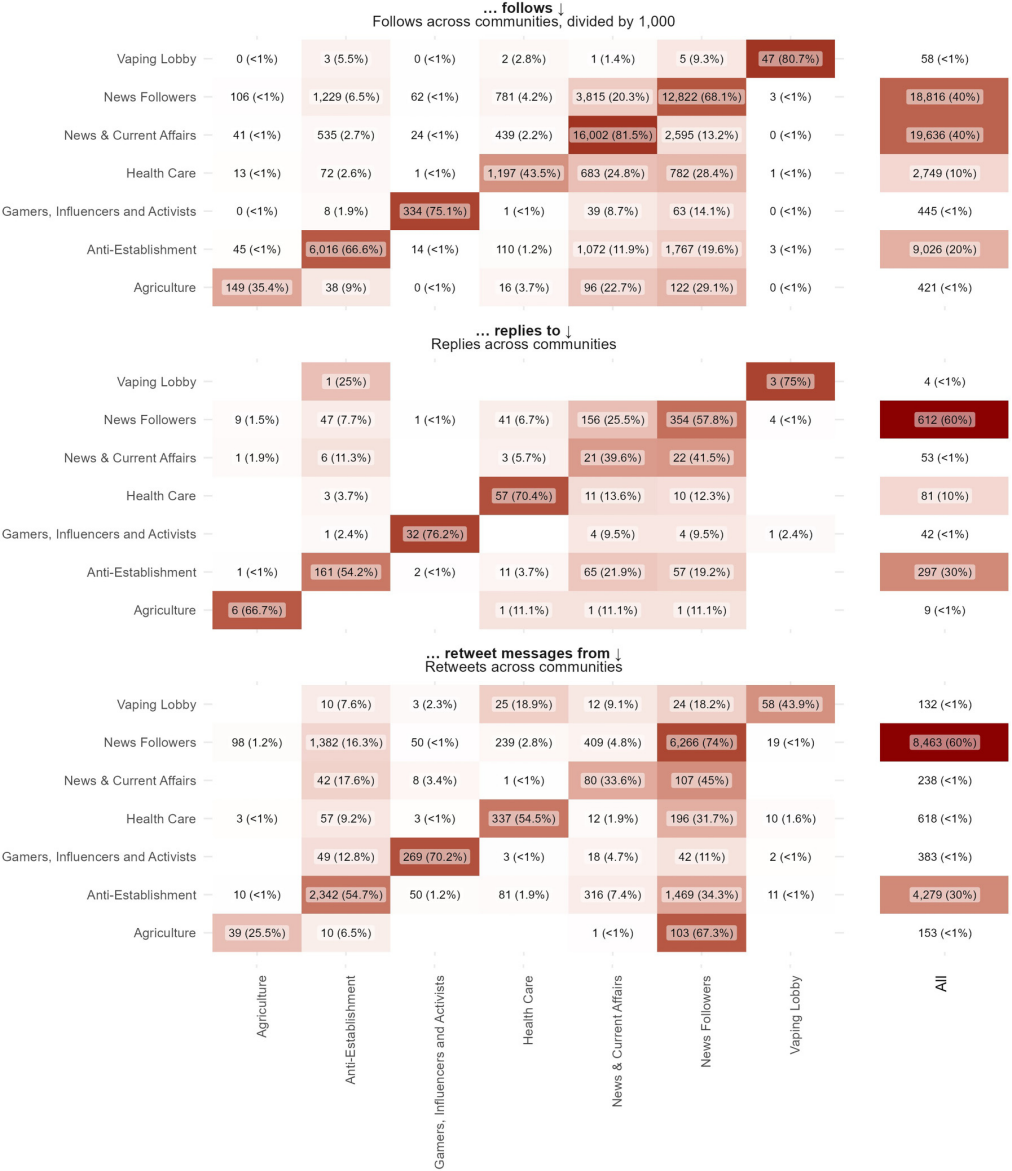
Absolute and relative engagement between communities.

Note: This figure shows the absolute and relative number of follows, replies, and retweets between communities.

Next, we computed the PageRank and Eigenvector Centrality scores for each Twitter user, reflecting their relative influence within the Twitter network.^36,38,39^ We used Gephi to visualize the networks,^40^ relying on its ForceAlas2 algorithm to lay out the graphs.^41^ The ForceAtlas2 algorithm places users who share many follower-followee relations close to each other (Figure 2). Note that the visualization of the Twitter network (i.e. Figure 2) does not explicitly show the follower relations that were taken into account by the ForceAtlas2 algorithm; rather, the links represent interactions between users such as retweets, quotes, and mentions. To analyze follower- and interaction patterns at the community level, we also created a heatmap (i.e. Figure 3).

#### Text mining and qualitative analysis to understand communities and conversations

We analyzed the tweets and profile descriptions for each of the communities. We created word clouds with word size expressing frequency, and color intensity expressing word salience (tf-idf).^42,43^ We also extracted hashtags used in tweets and created a hashtag network including the most frequently used hashtags, while linking hashtags that co-occur frequently. In the visualization (i.e. Supplemental Figure 1), the size of each hashtag signifies frequency, and the colors express prominence in each community. The figure includes the original hashtags in Dutch and have been translated into English in the results section.

The word clouds and hashtag network were used to analyze tweets and profile descriptions qualitatively, following the patterns that surfaced in the visuals to inspect the tweets and profiles that included specific words in their context. For example, when we noted that the word “vaping” was used relatively often in a certain community, we would retrieve all tweets including that word from the Neo4j database and analyzed the meaning of these tweets, the context in which the word was used, and what it told us about the community as well as their position in the wider debate. Communities were filtered for relevance for the Dutch setting. For example, we discarded a South-African and Flemish, community and made sure that the tweets, profiles, and relations were deleted from the Neo4j database.

## Results

Our initial search (tweets on the conversation level) returned 34,297 messages by 18,502 unique users. Retrieving the related retweets, quotes, and replies (tweets on the context level) resulted in 27,379 extra messages by 9289 unique users. By filtering for relevance by keywords, we removed 16,010 tweets and retained 24,699 messages by 14,330 unique users on the conversation level, and 20,967 messages by 7643 unique users on the context level. By retrieving the followers and followees of all active users (interests and audiences level), we retrieved 13,114,853 users of which the 10% best-connected users were retained. Table 3 shows the characteristics of our final data set after filtering and Figure 1 shows a flow chart of the process.

**Table 3.** Characteristics of the final Twitter data set.

Level	Tweets	Users	Average number of tweets per user	% Tweets	% Users
Conversation	24,699	14,330	1.72	54.1%	1.05%
Context	20,967	7643	2.74	45.9%	0.56%
Interests and audiences	0	1,336,634	0	0	98.39%

Note: The conversation level refers to the tweets we found using our search queries, the context level refers to the associated retweets, quotes, and replies, and the interest and audiences level corresponds to the 10% accounts that the other levels most commonly followed or were being followed by.

The Louvain algorithm that was used to detect communities of closely connected users returned a modularity score of 0.462, which signifies a relatively strong community structure. Table 4 shows the seven communities that were identified in the network, including the number of members and distinguishing between active (*conversation* and *context*) and inactive users (*interests & audiences*). The communities have been named based on the word clouds and qualitative analysis of the profile descriptions (see Supplemental Table 1).

**Table 4.** Characteristics of the Twitter network.

	Members		Conversation				Context							
			Members		Tweets		Members		Tweets		Interests & audiences		Average Number of Follows within the network	Average Number of Followers within the network
Community	N	%	N	%	N	%	N	%	N	%	N	%		
News & Current Affairs	749,437	63.8	370	<0.1	4547	13.9	400	0.1	4262	15.6	748,667	99.9	83.5	524
News Followers	258,543	22.0	6408	2.5	12,512	38.4	3689	1.4	12,801	46.7	248,446	96.1	156	139
Anti-Establishment	86,391	7.4	3659	4.2	7977	24.5	1283	1.5	7351	26,8	81,449	94.3	297	264
Health Care	54,034	4.6	780	1.4	5126	15.7	369	0.7	1662	6.1	52,885	97.9	125	131
Gamers, Influencers & Activists	15,637	1.3	1095	7.1	1411	4.3	272	1.7	707	2.6	14,265	91.2	17.6	14.1
Agriculture	9294	0.8	130	1.4	305	0.9	69	0.7	428	1.6	9095	97.9	199	106
Vaping Lobby	1965	0.2	85	4.3	728	2.2	14	0.7	176	0.6	1866	95.0	32.1	26.4

Note: The percentages of users in the conversation and/or context column show the percentage of the community members that published tweets about smoking and/or smoke-free policies. These percentages can be seen as indicators for relative involvement of each of the communities in the discourses on smoke-free policies.

Figure 2 shows a visualization of the network. The community *News & Current Affairs* is the largest community but only accounts for a relatively small proportion of the conversations on Twitter. In absolute terms, the *News Followers* community most frequently tweeted about smoke-free policies (51.2% of all tweets), followed by the *Anti-Establishement* community (29.2%), and *Gamers, Influencers, and Activists* community (8.7%). The *Health Care* community accounted for 5.2% of the Twitter messages. Table 4 shows these figures while distinguishing between messages on the conversation and context levels.

### Communities

In the following paragraphs, we describe the communities in more detail, probe their level of expressed support for novel smoke-free policies, and map their alignment in the wider debate. The descriptions are based on the word clouds, hashtag network, and qualitative analysis of the tweets (see Supplemental Table 1 and Supplemental Figure 1).

#### News followers

The *News Followers* community is a large cluster of Twitter users who are mostly from the Netherlands and who mostly support novel smoke-free policies. The community's members use their Twitter accounts for personal purposes, sharing their views and ideas about news and public opinion about all kinds of topics, including smoking and smoke-free policies. In the profile descriptions, the members describe themselves as mothers or fathers and mention their job title and their hobbies. The community is closely connected with the *News & Current Affairs* community, suggesting that, combined, the two communities mostly engage with news and topics that are raised by the traditional Dutch (news) media, such as newspapers and broadcasting organizations.

The *News Followers* community is mostly engaged in debate about news and current affairs, including smoking and smoke-free policies. The most prevalent hashtags used by the *News Followers* community refer to political parties and politicians, news magazines and TV shows, and topics such as air pollution, drug policies, climate change, and COVID-19 measures. The only hashtag that specifically touches the subject of smoke-free policies is “smokingbanonspanishbeaches”: a topic that seems to upset some of the members of the *News Followers* community, arguing that a smoking ban on beaches is excessive.

#### Anti-establishment

Members of the *Anti-Establishment* community mostly describe themselves as “critical citizens” and often sympathize with right-wing and anti-establishment parties and ideologies. In their profile descriptions, the members specifically mention what they hate and what they are against. Compared to the active community members (i.e. users that have published tweets about smoking and/or smoke-free policies), the inactive community members (i.e. the interests and audiences that the active users have in common) seem to be more internationally oriented (meaning that they often tweet in English and/or that their profile descriptions are in English) and include entrepreneurs and crypto experts.

Members of the *Anti-Establishment* do not seem to be too interested in conversations about smoke-free policies. When they do refer to such policies, it is often in the context of agitating against what they see as “repression.” Hashtags such as “car,” “sugar,” “meat,” “obligatorymask,” “idontwantavaccine,” “freedom” and “stopthelockdown” surface almost exclusively from the *Anti-Establishment* community and are retweeted a lot within this group. For example, the following tweet was retweeted 52 times, of which 46 times by members of the Anti-Establishment community: “Thou shalt not eat #fat, #sugar, #meat, drink #alcohol, #smoke, or drive a #car or #fly.”^a^

The Anti-Establishment community interacts frequently with the *News Followers* community, but these interactions mainly focus on the COVID-19 measures taken in the Netherlands. They surface in our data set because the association with “other repressive legislation” is repeatedly made by members of the *Anti-Establishment* community.

#### Gamers, influencers & activists

The *Gamers, Influencers & Activists* community is a mixed community of young activists and social media-, music- and gaming enthusiasts. In their tweets, three words stand out with high tf-idf scores (i.e. balcony, simply, and joint), highlighting a large number of messages in which the users announce that they are or feel like smoking a cigarette or joint in the garden or balcony. These messages do not really tie into the larger debate on smoke-free policies and also in terms of connectedness the members of this community stand at some distance from the rest of the network.

#### Health care

Active members of the Health Care community include public health professionals such as general practitioners, medical doctors, nurses, academics, and prevention experts. The inactive members in this community describe themselves as nurses, employees of public health services, and health advisors.

The *Health Care* community dominates the debate on hashtags such as “smoking,” “lifestyle,” “healthcare,” “quitsmoking,” “cigarette” and other hashtags with a direct relation to the topic of smoking and smoke-free policies. For example, the community promotes “stoptober” (the name of an annual campaign motivating smokers to quit their habit in October) and exchanges experiences about quitting smoking with the *News Followers* community; defends measure to raise taxes on tobacco (“tobaccotaxes”), calling it the most effective way to prevent young people from starting to smoke; and shares medical information about the impact of smoking (“bloodpressure,” “cancer,” “copd”).

At the same time, members of the *Health Care* community actively warn against the influence of the tobacco lobby. A prominent anti-smoking activist is particularly active in this respect, using hashtags such as “lobbycracy” (a wordplay on lobby and democracy) and attracting many retweets from within the *Health Care* and *News Followers* community. Activists within this community also point out which Twitter users work for the tobacco lobby, calling them trolls that spread misinformation. In some instances the tone is harsh and/or condescending, using hashtags such as “lungcancerisachoice” and “fatandstupid.” Hardly any of these messages are retweeted or responded to beyond the *Health Care* community.

#### News & current affairs

The active accounts in the News & Current Affairs community mostly concern the editorial staff of news platforms and TV and radio shows, alarm services, and web care accounts of companies. The tweets mostly concern links to news articles or videos, and hardly ever concern interactions such as replies. The community only occasionally gets involved in debates about smoke-free policies. The inactive accounts are mostly followers of these news accounts.

Despite being the largest community of Twitter users, the *News & Current Affairs* community plays a marginal role in the discourse on smoke-free environments. The most frequently used hashtags in this community are the names of popular talk shows and news shows on Dutch TV, and “health”—a hashtag that is also commonly used by the *Health Care* community. In the wider debate, the *News & Current Affairs* mainly plays the role of news and information disseminator.

#### Agriculture

The Agriculture community includes farmers, researchers, politicians, and lobbyists. In the context of the debate about smoke-free policies, the community mostly compares smoke-free policies with higher taxation of meat—often in discussions with members of the anti-establishment community about “other repressive measures” (i.e. taxation of gasoline, COVID-19 measures, taxation on flights, etc.). The Agriculture community is hardly interested in discussing smoke-free environments but does agitate against what they see as repressive legislation and often interacts with the Anti-Establishment community.

#### Vaping lobby

The *Vaping Lobby* community includes accounts from what appear to be lobbyists of the tobacco industry, news platforms, and consumer groups. In the word clouds, we see that their profile descriptions and tweets stand out as they specifically mention words such as “dampen”/“dampers” (vaping/vapers), “sigaret” (cigarette), “vapen” (to vape), and “schadelijk” (harmful). The community clearly advocates for looser legislation on vaping, presenting it as a healthier alternative to smoking and more restrictive legislation as harmful to public health.

The *Vaping Lobby* community most frequently uses hashtags such as “tastenecessity,” “vaping,” “ecigarette,” and “vapingsaveslives.” Members of the vaping lobby often refer to websites (e.g. R Street Institute, RealClearPolicy) or videos (e.g. https://www.youtube.com/watch?v = 2e_14baQt80) that look like legitimate news outlets but appear to be funded by groups that are lobbying for a smaller, less invasive government. The links often function as “proof” for claims such as that smokers switching to vaping would result in the same public health gains as smokers quitting altogether, or that a ban on flavored vapes would have unintended negative public health consequences. The *Vaping Lobby* community also asks conservative parties for their stance on vaping, possibly to provoke a debate between politicians on the issue. The lobby also refers to a petition to stop the intended ban on flavored e-cigarettes in the Netherlands, which has gotten some traction among the *Anti-Establishment* community, especially among people who claim to have successfully quit smoking with the help of vaping products. Another strategy of the *Vaping Lobby* is to respond and counter messages published by members of the *Health Care* community.

### Interactions between communities

We explored how the communities followed and engaged with each other on the community level to get a better sense of the public debate's dynamics (see Figure 3).

#### Follows

Table 4 and Figure 3 show that the number of follows and followers vary greatly across communities, with the *News & Current Affairs* having the highest average number of followers (Table 2) and total follows (Figure 3) within the network, followed by the *News Followers* and *Anti-Establishment* communities. Figure 3 shows that all communities mostly follow members from within their communities, although to varying extents: the *News Followers*, *Health Care and Agriculture* communities follow members from other communities relatively often, while the *Anti-*Establishment, *News & Current* Affairs, and *Vaping Lobby* are more likely to follow within their community.

#### Replies

The *News Followers* community has published the most replies, followed by the *Anti-Establishment* community; and the *News Followers* and *News & Current Affairs* communities are relatively most replied to. Figure 3 shows that the *News Followers* and *Anti-Establishment* mostly direct their replies to the *News & Current Affairs* community, with the number of responses remaining limited. However, the *News & Current Affairs* community does seem to have a preference for responding to the *News Followers* community, with much less replies directed to the *Anti Establishment* community.

#### Retweets

The *News Followers* community retweets most frequently and is retweeted by others most frequently, thereby appearing to play an important role in disseminating news. The community mostly retweets its own messages, followed by messages by the *News & Current Affairs* community and the *Health Care* community. The *Anti-*Establishment community frequently retweets too, mostly from itself, the *News Followers* and the *New & Current Affairs* community. Strikingly: they appear to retweet the Health Care community relatively little. Although the Health Care community is not exactly retweeted often, they appear to play an important role in providing health information to the News Followers community. Strikingly, the *Vaping Lobby* is hardly retweeted by other communities.

## Discussion

Although large communities such as *News Followers*, News & *Current Affairs,* and *Anti-Establishment* published the highest absolute number of messages, our study shows that the smaller *Health Care, Vaping Lobby*, and *Agriculture* communities appear to be the most substantively engaged in relative terms. Each of the communities appeared to discuss smoke-free policies from its own perspective: the *Health Care* community focused on spreading health information and exposing the tobacco lobby; the *Vaping Lobby* actively pushed vaping as a healthy alternative to smoking; and the *Anti-Establishment* community discussed smoke-free policies in the context of increasing oppression of civil liberties. The *Health Care* community seems to have succeeded in raising awareness of lobbying and misleading practices by the tobacco industry. Their messages that expose the tobacco lobby are discussed by other communities and featured in popular TV programs.

Our study also suggests that opposition against novel smoke-free policies can be seen in the context of the increasing prevalence of (health) disinformation and anti-institutional sentiment on Twitter, which has further increased after the take-over by Elon Musk and Twitter's rebranding to X. Previously, this has been linked to a growing distrust in traditional institutions such as media and (medical) science: a pattern that has surfaced in debates about vaccination, the COVID pandemic and political polarization.^11,28,44–46^ Previous studies have established that the systematic spread of misinformation can be weaponized by tapping into such sentiment, potentially increasing distrust in traditional institutions.^26^ If the *Vaping Lobby* were to align its argumentation with narratives dominant in the *Anti-Establishment* community (e.g. “governments infringe personal liberties,” “science is rigged,” “the elite is after the repression of the common man”) vaping could become the next linking pin in a large web of conspiracies that seems to energize the *Anti-Establishment* community.

### Limitations

A clear limitation is that the study solely focuses on Twitter that, at that time, was mostly used by journalists, politicians, communications professionals, scientists, and avid news followers, which may have biased findings to these groups. Furthermore, Dutch citizens can be found on other social media platforms too that were not included. This study cannot be seen as representative of all public discourse on smoke-free policies, neither for online discourse on the topic: it solely focuses on Dutch Twitter. This also means that the findings only apply to the Dutch context that, when data collection took place, was hit by the COVID-19 pandemic. This resulted in finding many tweets that mentioned smoke-free policies as an example of “repression,” while being primarily about the COVID measures.

Although the public debate on Twitter may not be representative of wider discourses across the internet, a strength of the approach presented in this study is that it disentangles the voices of various communities, mapping their opinions, relationships, and preferred sources, which can help in better addressing factors related to public support when implementing new smoke-free policies as are part of socio-ecological approaches.^22^ After all, Twitter does play a role at the backdrop of the implementation of smoke-free policies and the public debate about it.

## Conclusion

This study identified the most discussed themes related to smoke-free policies on Twitter. Among general audiences, new policies such as changes in conditions of how tobacco can be sold, smoke-free hospitals, university campuses, and beaches were among the most discussed news events.^26^ This study also mapped the alignment of various online communities in the discourse about smoke-free policies. Except for the *Vaping Lobby* and various members of the *Anti-Establishment* communities, there seems to be support for implementing novel smoke-free policies. However, if the *Vaping Lobby* were to align its argumentation with the *Anti-Establishment* community, vaping could become the next linking pin in a large web of anti-institutional conspiracies, spill over to places beyond X, and frustrate the implementation of novel smoke-free policies. This study can inform future work to understand how online discourses shape public support for health policies. Further research is needed to identify the ways in which social media shapes public support, how the spread of misinformation can be prevented, and how social media platforms can be leveraged to involve civil societies in policy-making.

## Supplemental Material

sj-docx-1-dhj-10.1177_20552076251325583 - Supplemental material for Discourses on smoke-free policies on Dutch Twitter: A social network analysis Supplemental material, sj-docx-1-dhj-10.1177_20552076251325583 for Discourses on smoke-free policies on Dutch Twitter: A social network analysis by Roel O Lutkenhaus, Abel Meijberg, Famke JM Mölenberg, Jasper V Been and Martine PA Bouman in DIGITAL HEALTH

## notes

a. This tweet was translated and paraphrased to prevent identification of its author.

b. Dutch for ‘smoke-free’ and ‘tobacco-free’.

c. Dutch for ‘smoke’ and ‘tobacco’, and synomyms and slang for ‘cigarette’ and ‘to smoke’, ‘cigar(s)’, ‘smoker’ and ‘rolling tobacco’.

d. Dutch for ‘policy’, ‘rule’, ‘to regulate’, ‘laws’, ‘legislation’, ‘prohibition’, ‘to forbid’, ‘you cannot’, ‘illegal’, ‘punishable’, ‘fine’, ‘excise duty’, synonyms for ‘costs’, ‘to discourage’, ‘to limit’ and ‘measure’.

e. Dutch for ‘zone’, ‘area’, ‘bus stop’, ‘school’, ‘environment’, ‘terrain’, ‘hospital’, ‘public space’, ‘terrace’, ‘restaurant’, ‘car’, ‘house’, ‘home’, ‘apartment’, ‘balcony’, ‘bar’, ‘beach’, ‘airport’ and ‘train station’.

f. Dutch for ‘government’, ‘child’, ‘youth’, ‘junior’, ‘toddler’, ‘baby’, ‘minor’, ‘parent’, ‘staff’, and ‘employee’.

g. Dutch for ‘welfare’, ‘health’, ‘development’ and ‘clean air’.

h. Dutch for ‘support’, ‘opinion’, ‘public opinion’, ‘nuisance’, ‘rights’, ‘autonomy’, ‘free choice’, ‘patronizing’ and ‘at odds with’.
