# Supplementary material for: Discourses on smoke-free policies on Dutch Twitter: A social network analysis
Source: Digit Health. 2025 Mar 14;11:20552076251325583. doi: 10.1177/20552076251325583 (PMC11909667; doi:10.1177/20552076251325583)
Supplement: sj-docx-1-dhj-10.1177_20552076251325583 - Supplemental material for Discourses on smoke-free policies on Dutch Twitter: A social network analysis [file sj-docx-1-dhj-10.1177_20552076251325583.docx]

# Supplement

**Supplemental Table 1.**

Word clouds for the profile description and tweets by the users in the network

| **Community** | **Profile descriptions of active users (conversation and context)** | **Profile descriptions of inactive users Inactive (interests and audiences)** | **Tweets** |
| --- | --- | --- | --- |
| News & Current Affairs | 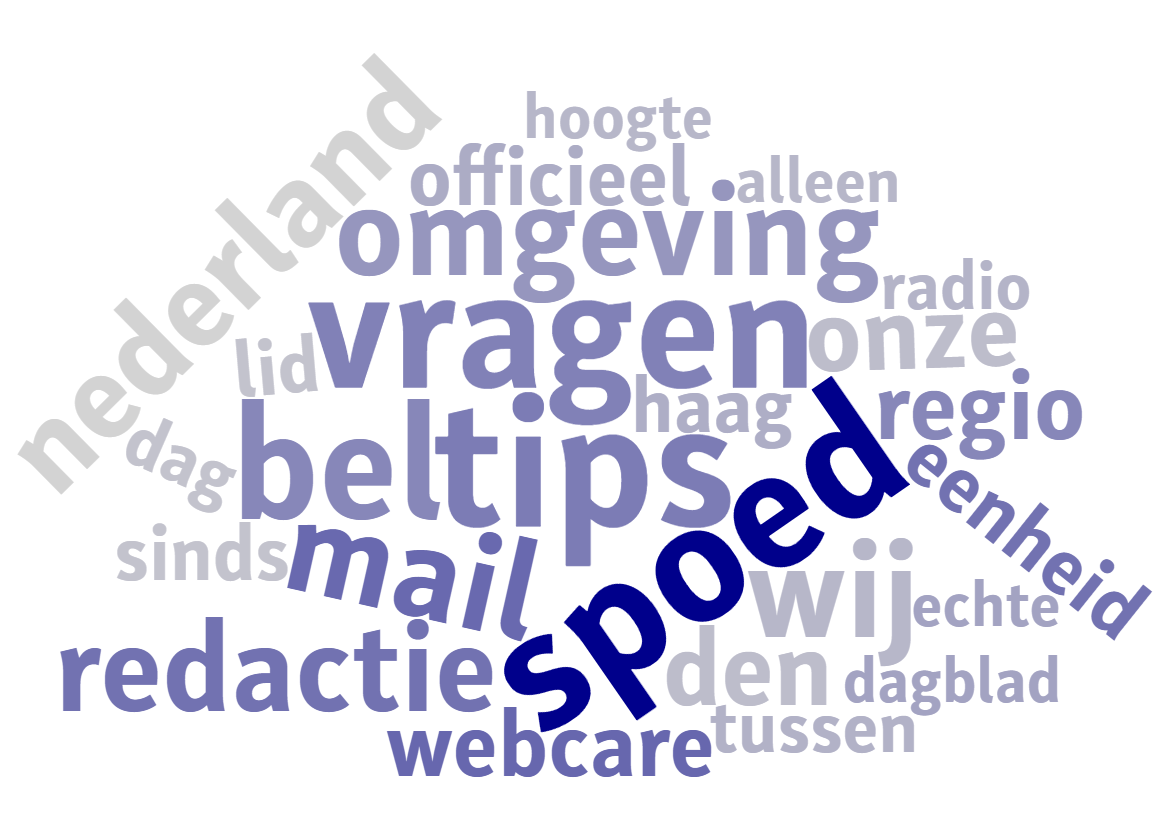 | 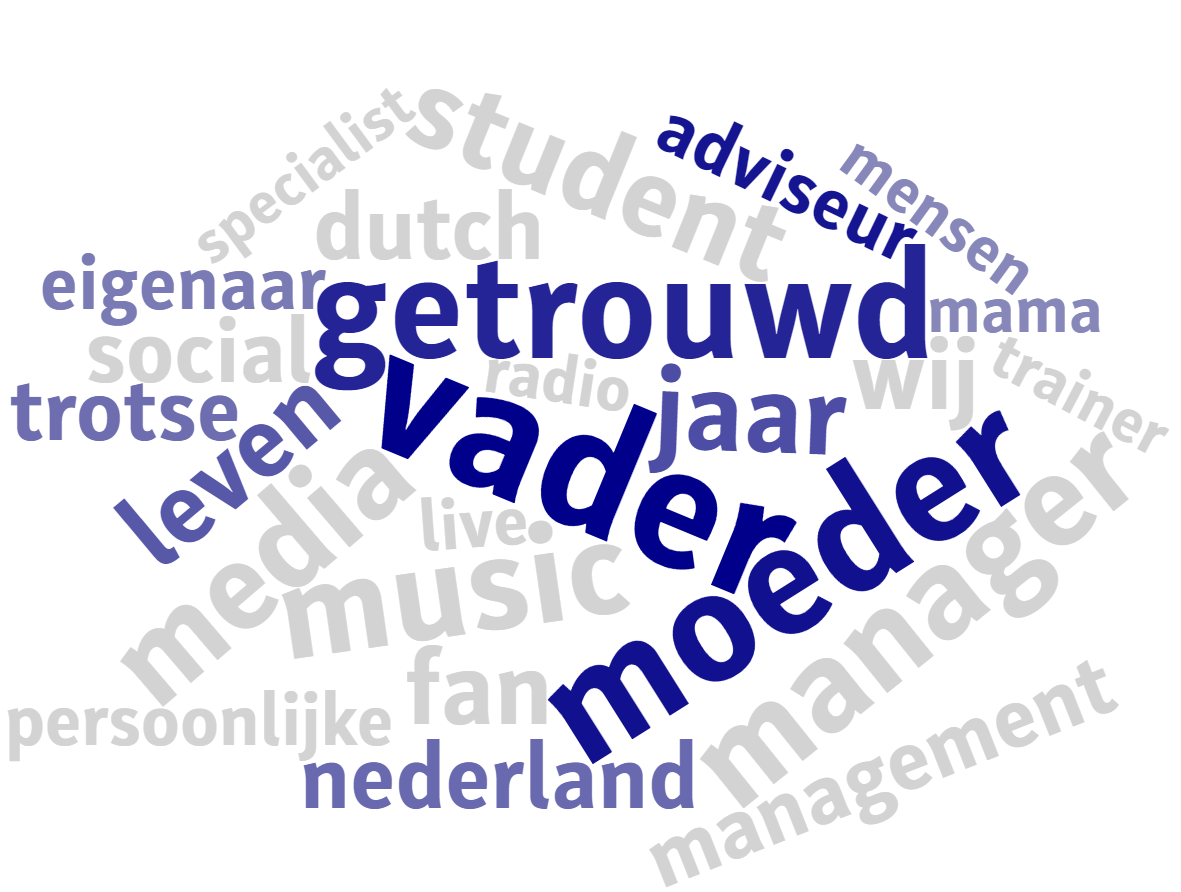 | 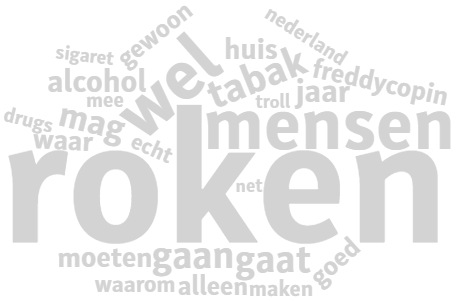 |
|  | tips, emergency, to ask, call, mail, we, environment, the netherlands, editorial, the, our, region, web care, unit, officially, hedge, member, since, day, between, radio, only, daily newspaper, real, height | father, mother, married, manager, music, media, student, year, to live, fan, we, dutch, the netherlands, social, proud, management, owner, advisor, personal, live, mom, people, radio, trainer, specialist | smoking, people, well, tobacco, to go, go, may, year, alcohol, house, must, where, freddycopin, good, only, ordinary, why, real, to make, with, the netherlands, drugs, cigarette, just, troll |
| *News Followers* | 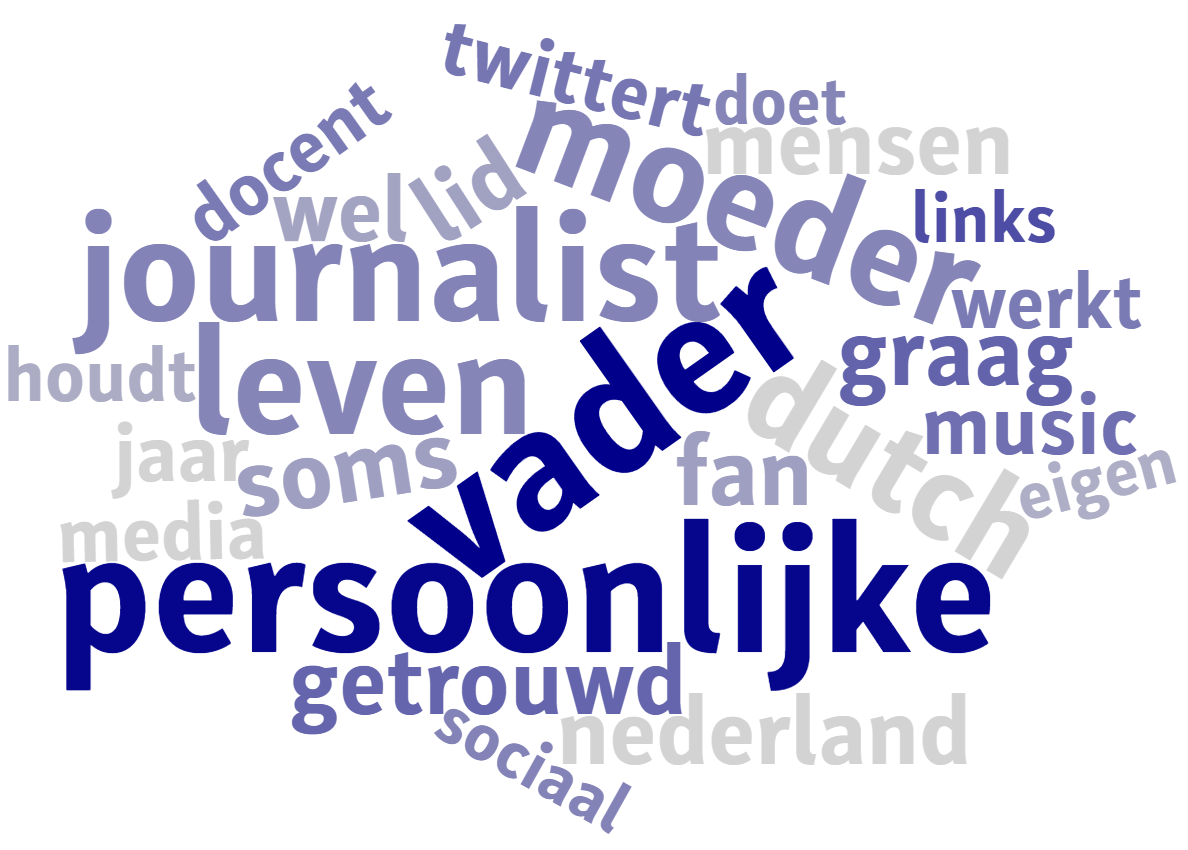 | 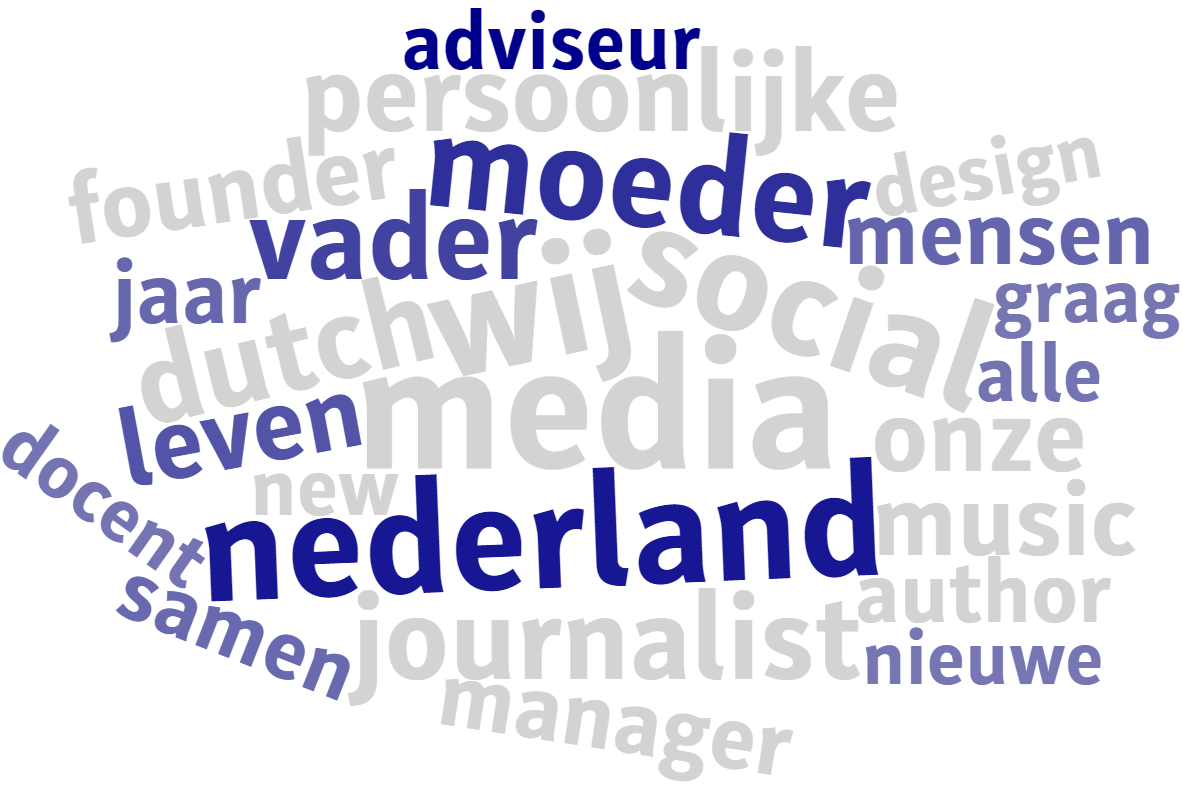 | 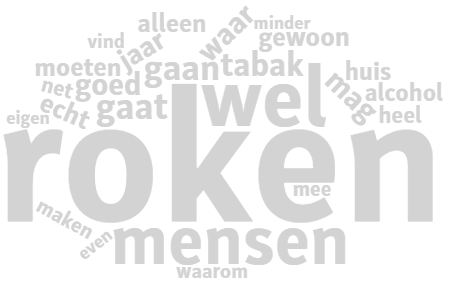 |
|  | father, personal, journalist, to live, mother, dutch, sometimes, fan, please, married, member, the netherlands, people, well, music, year, tweets, media, lecturer, works, holds, left, own, social, do | media, we, the netherlands, social, mother, dutch, journalist, father, personal, to live, our, music, founder, manager, people, year, author, together, new, design, advisor, all, lecturer, new, please | smoking, well, people, go, to go, tobacco, may, good, where, year, real, ordinary, must, only, house, alcohol, all, just, to make, with, why, find, own, even, fewer |
| Anti- Establishment | 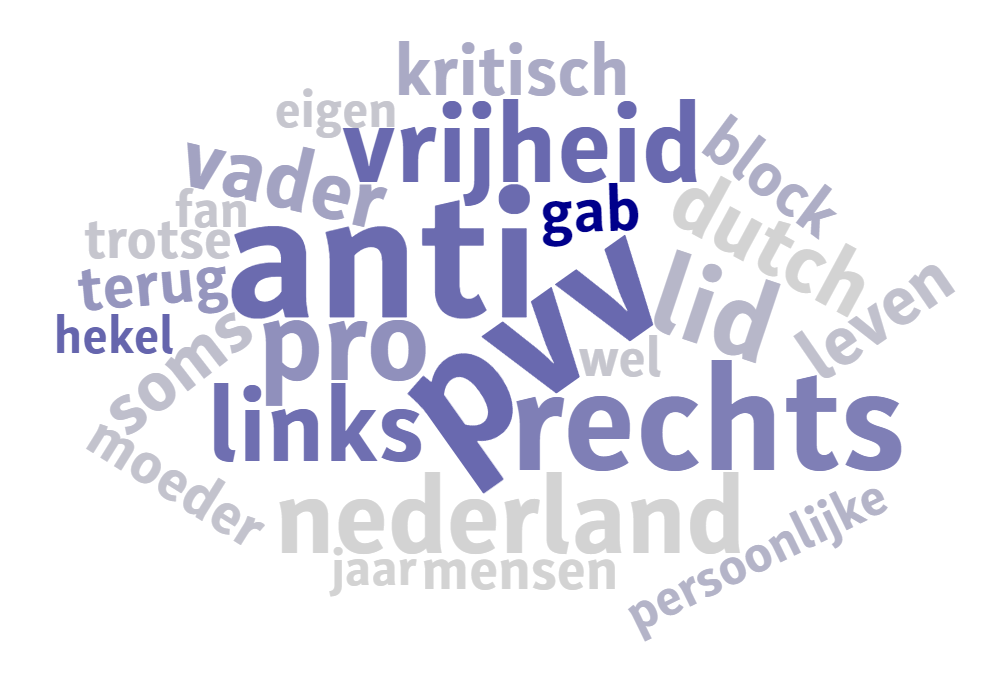 | 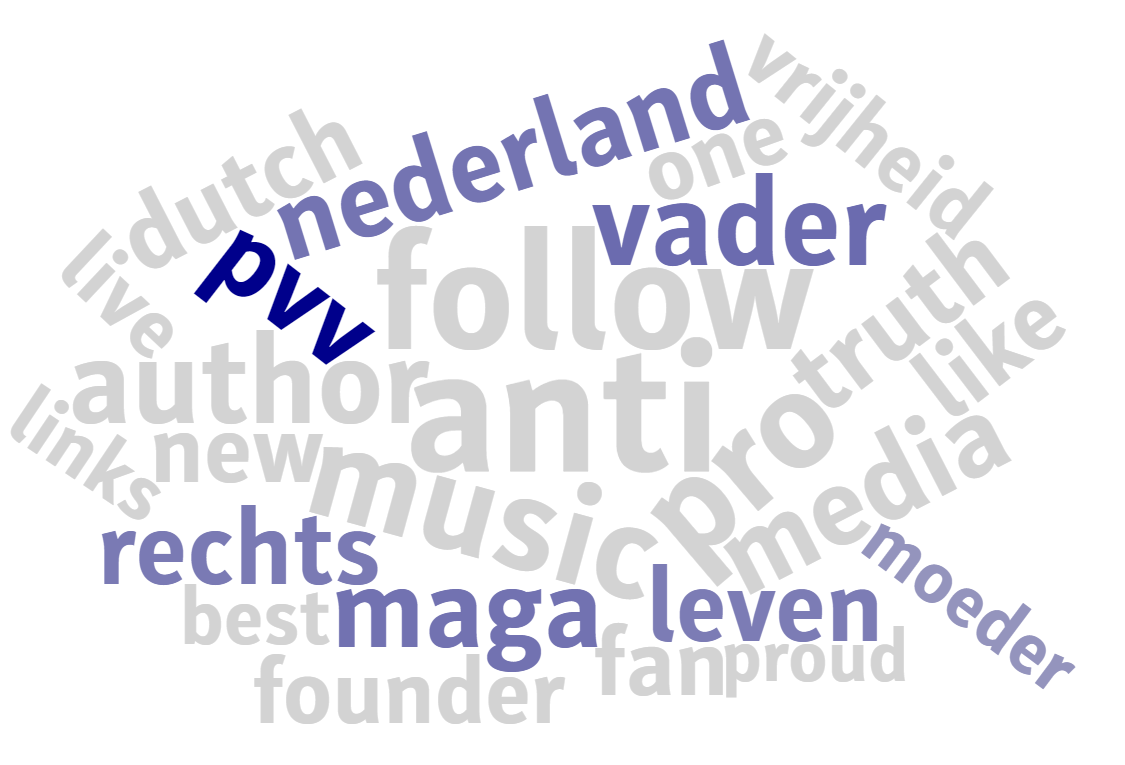 | 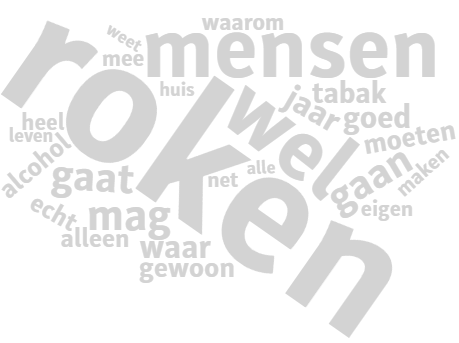 |
|  | pvv, anti, right, pro, member, freedom, the netherlands, left, dutch, father, sometimes, to live, critical, block, back, gab, mother, people, proud, well, personal, own, fan, hate, year | anti, follow, pro, music, author, father, media, maga, pvv, the netherlands, truth, right, to live, dutch, like, new, fan, founder, one, live, freedom, left, best, mother, proud | smoking, well, people, may, go, to go, year, where, good, tobacco, ordinary, must, alcohol, only, real, why, with, all, own, to make, just, house, to live, all, know |
| Health Care | 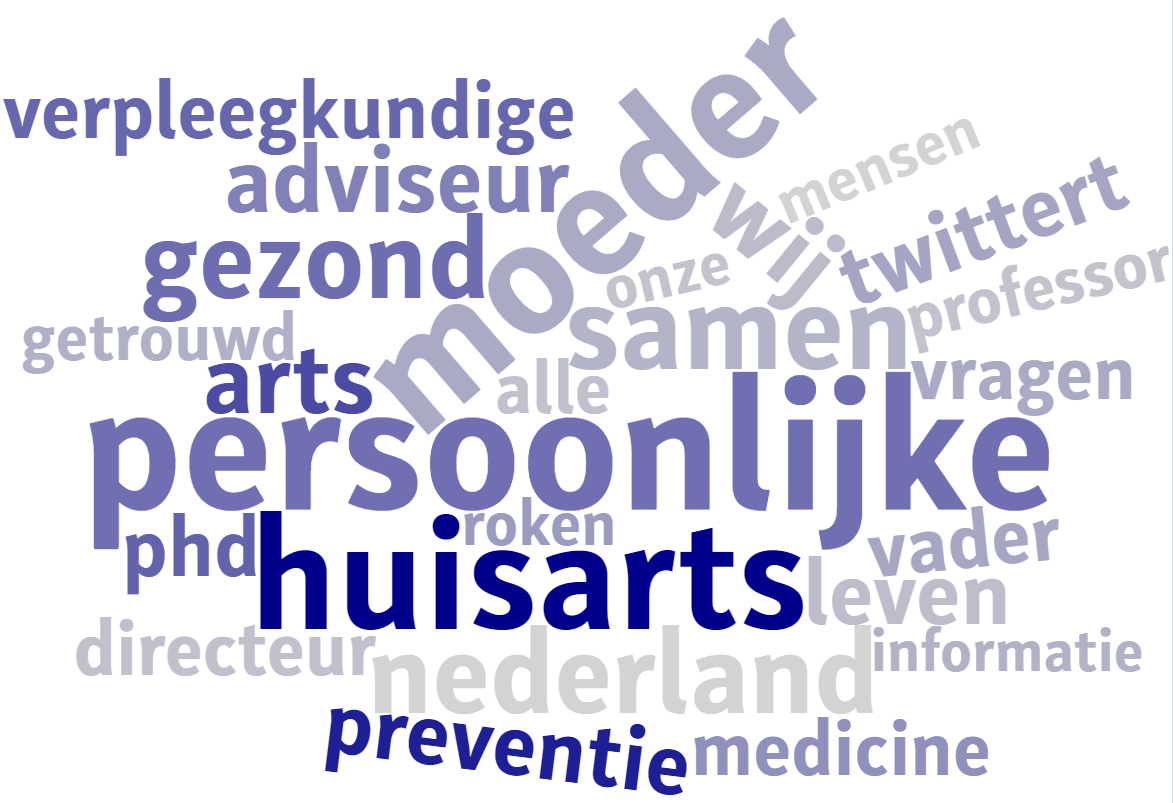 | 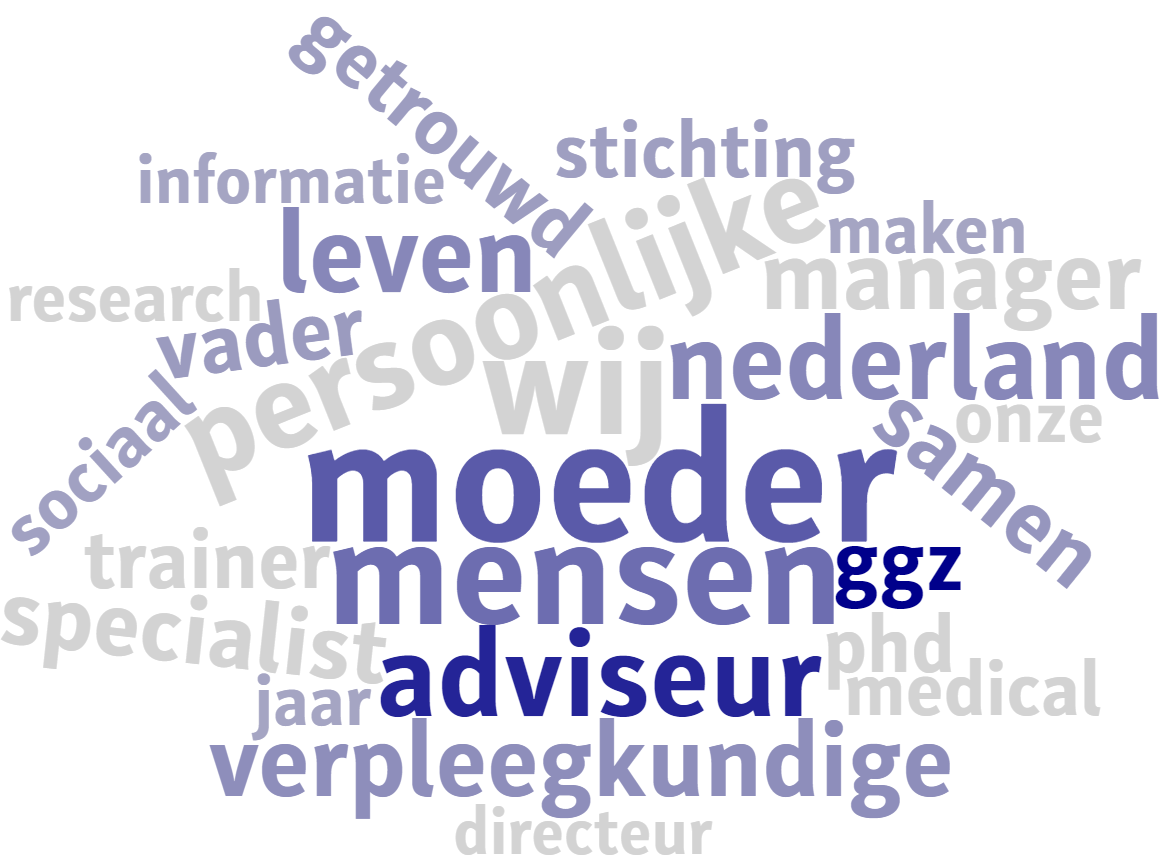 | 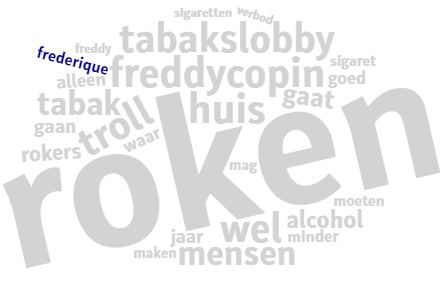 |
|  | personal, mother, gp, together, the netherlands, healthy, we, doctor, advisor, to live, prevention, tweets, phd, father, nurse, director, medicine, to ask, all, married, professor, people, our, smoking, information | mother, people, we, personal, advisor, the netherlands, to live, nurse, manager, specialist, together, mental health care, father, trainer, married, phd, founding, medical, social, our, year, to make, research, information, director | smoking, freddycopin, house, troll, well, tobacco lobby, people, tobacco, go, alcohol, smokers, to go, year, only, good, where, fewer, frederique, must, cigarette, may, to make, freddy, cigarettes, ban |
| Gamers, Influencers & Activists | 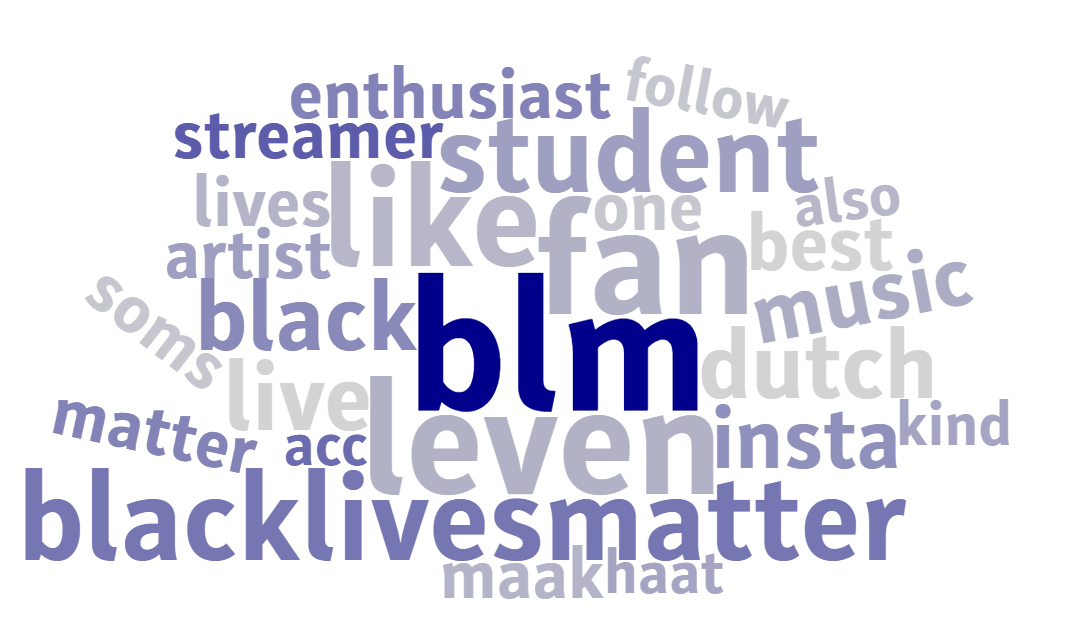 | 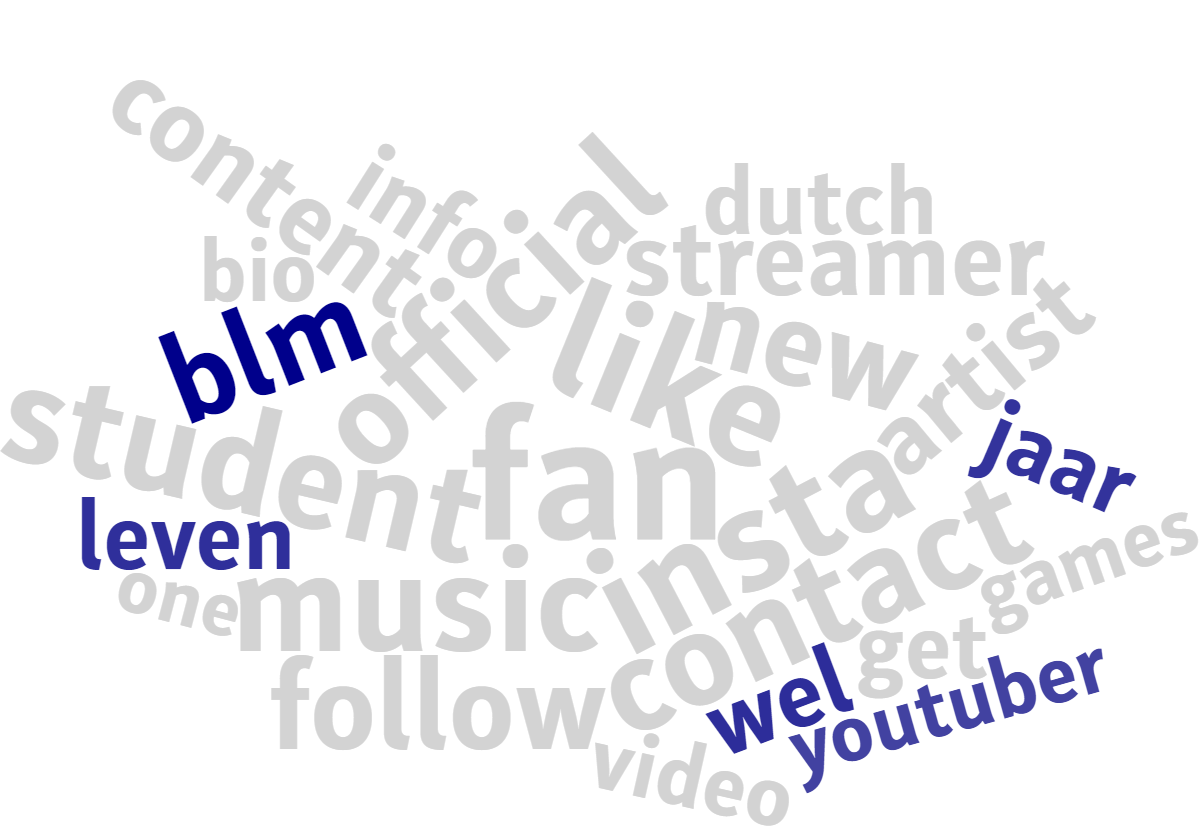 | 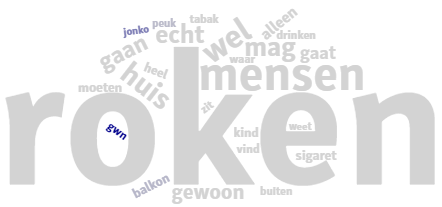 |
|  | blm, fan, to live, like, blacklivesmatter, student, black, dutch, live, insta, music, best, artist, enthusiast, lives, make, matter, one, sometimes, streamer, acc, also, follow, hate, child | fan, like, insta, music, student, contact, official, new, follow, blm, streamer, content, artist, information, well, to live, dutch, get, year, video, bio, blacklivesmatter, youtuber, games, one | smoking, people, well, house, may, real, to go, ordinary, go, only, cigarette, balcony, must, child, all, find, drinks, gwn, where, tobacco, outside, sit, cigarette butt, young, know |
| Agriculture | 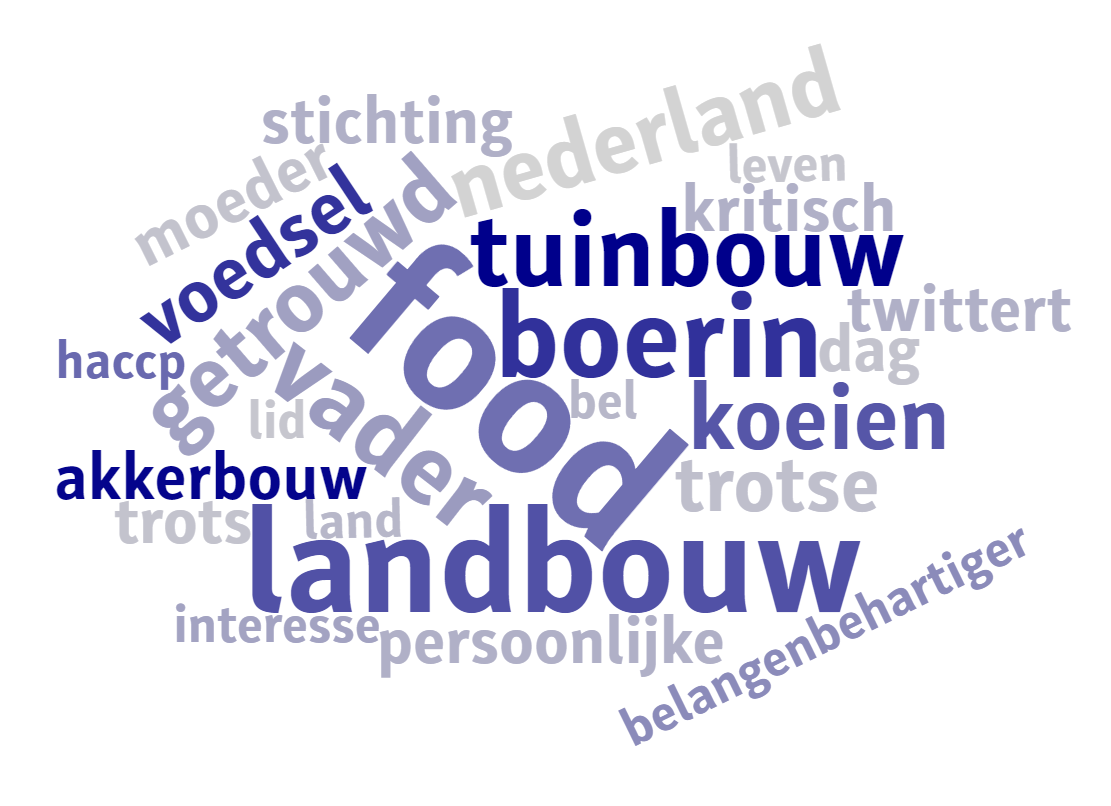 | 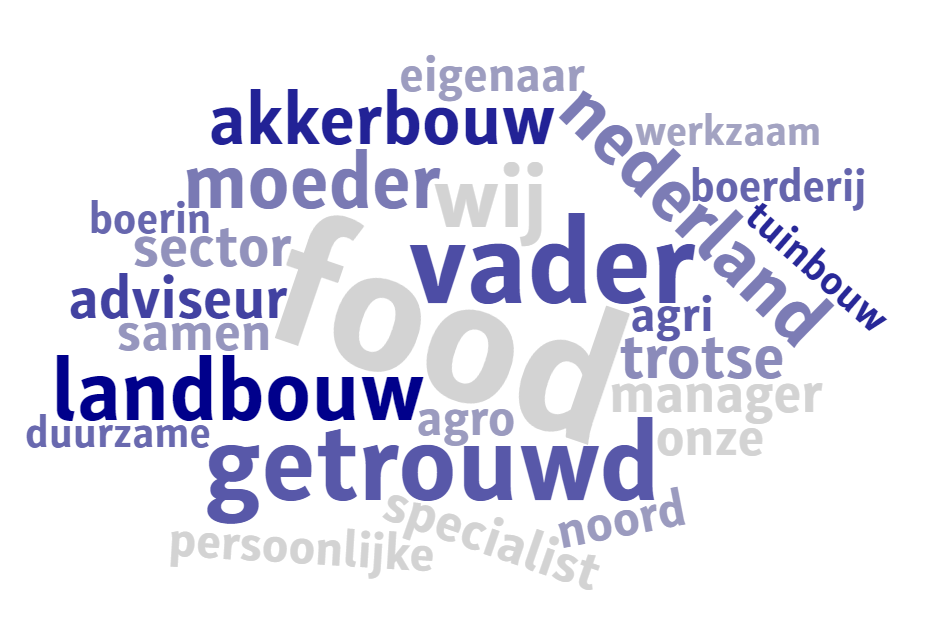 | 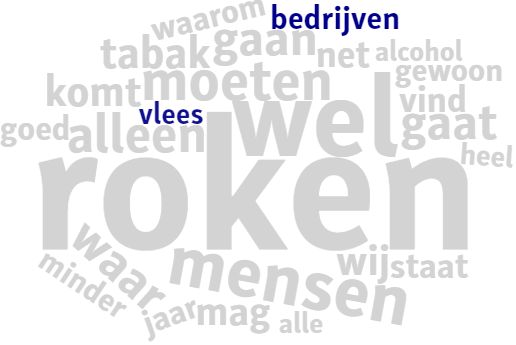 |
|  | food, agriculture, farmer, horticulture, father, married, cows, the netherlands, proud, food, arable farming, day, critical, mother, personal, founding, proud, tweets, call, advocate, haccp, interest, country, to live, member | food, father, married, we, agriculture, mother, the netherlands, arable farming, proud, advisor, sector, manager, together, specialist, our, agriculture, north, personal, agriculture, owner, farm, farmer, sustainable, working, horticulture | smoking, well, people, must, only, where, to go, go, tobacco, comes, we, may, just, find, stands, good, year, why, companies, ordinary, fewer, all, meat, alcohol, all |
| Vaping Lobby | 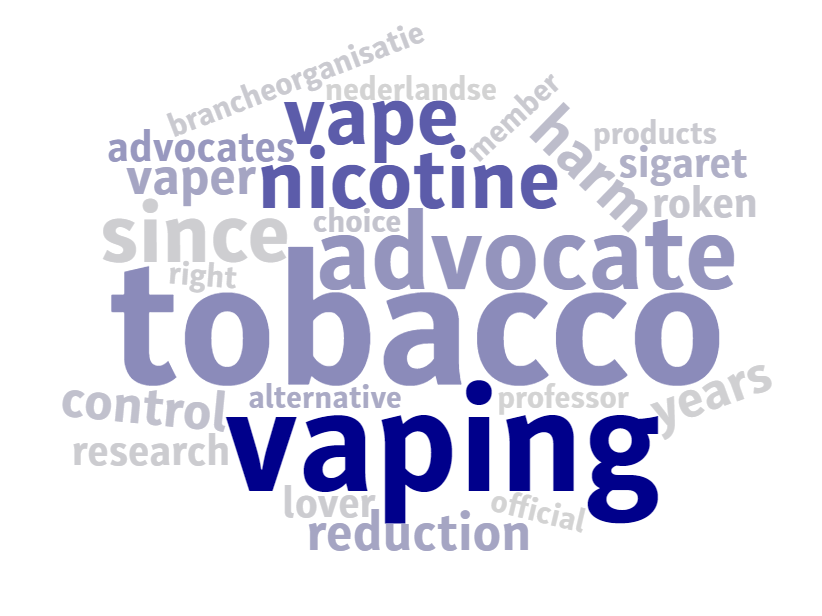 | 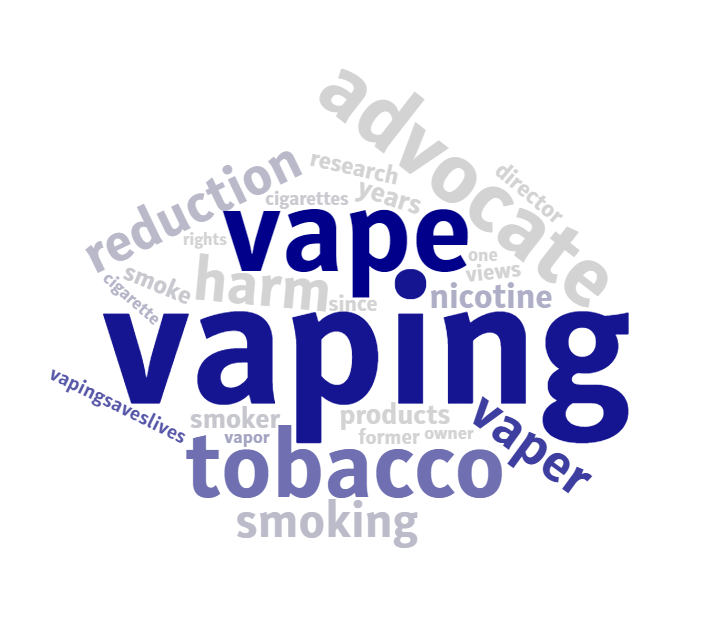 | 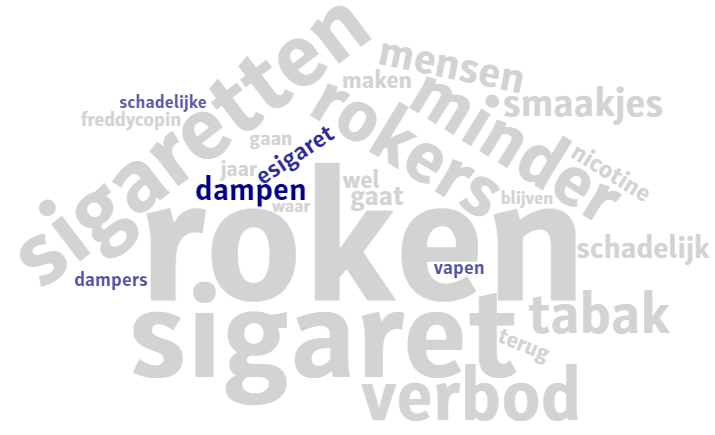 |
|  | tobacco, vaping, advocate, nicotine, since, vape, harm, control, reduction, vape, years, advocates, foliage, research, smoking, cigarette, alternative, trade association, choice, member, dutch, official, products, professor, right | vaping, vape, tobacco, lawyer, harm, vape, reduction, tuxedo, nicotine, products, smoker, years, smoke, research, director, views, since, former, vapingsaveslives, cigarettes, one, owner, vapor, cigarette, rights | smoking, cigarette, cigarettes, smokers, fewer, ban, tobacco, people, flavours, vapors, harmful, go, e-cigarette, nicotine, to make, well, back, year, to go, vaporizers, freddycopin, vaping, to stay, harmful, where |

*Note.* The wordclouds show the words that most frequently occur in the profile descriptions and tweets of the members of each community. Word size corresponds to frequency, whereas the color intensity corresponds with the extent to which a word occurs frequently compared to other communities. The translations are provided below, in order of word frequency.

**Supplemental Figure 1.**

The hashtag network.


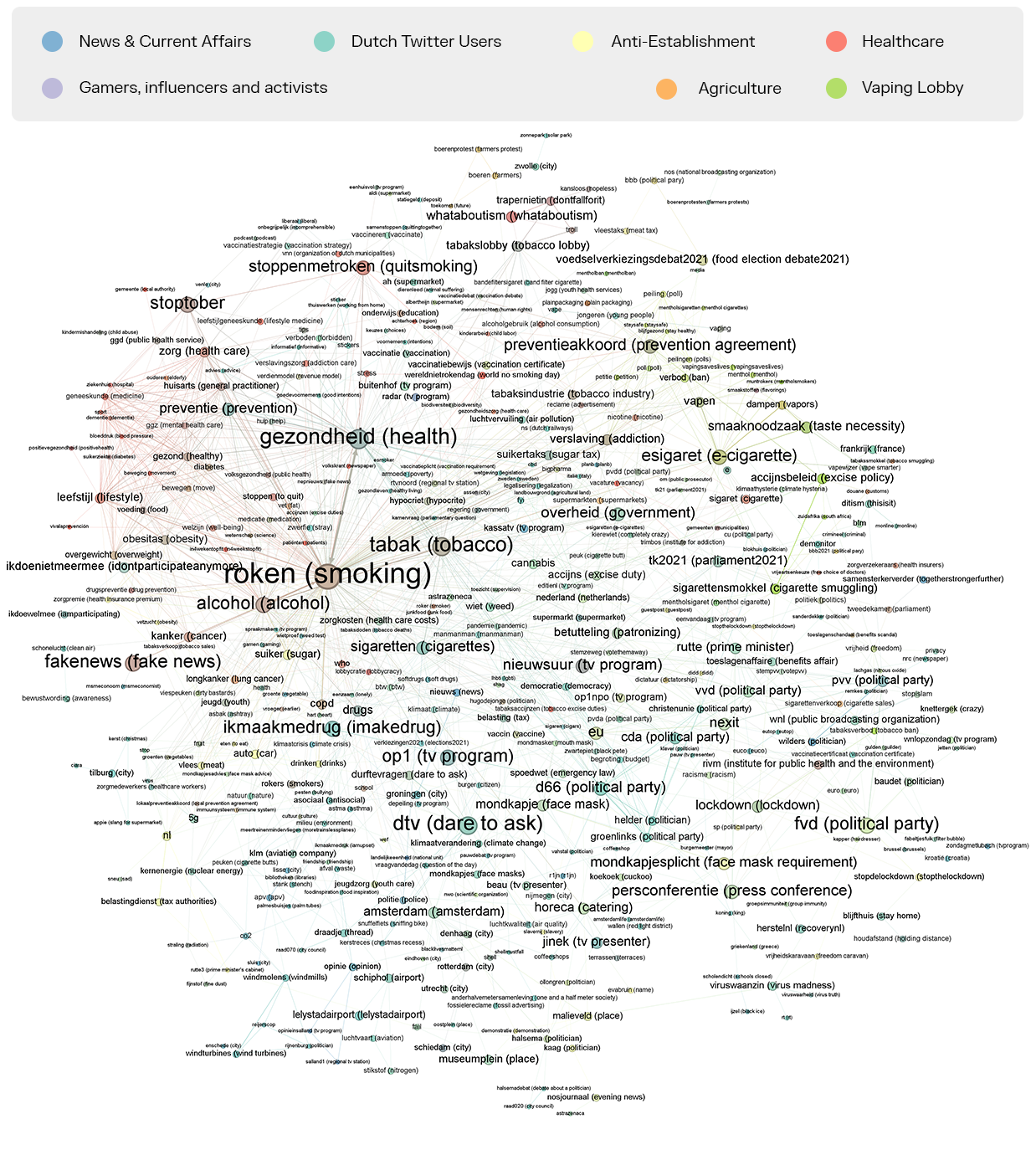


Note. The dots signify hashtags and their sizes express their frequency across the dataset. The lines between hashtags signify how often the hashtags have co-occurred. The colors of the dots express in which communities the hashtags occurred, where the colors of the hashtags that occurred in more than one community have been mixed to reflect the proportions.
